# The Expanding Role of MT1-MMP in Cancer Progression

**DOI:** 10.3390/ph12020077

**Published:** 2019-05-20

**Authors:** Anna M. Knapinska, Gregg B. Fields

**Affiliations:** 1 Department of Chemistry & Biochemistry and the Center for Molecular Biology & Biotechnology, Florida Atlantic University, Jupiter, FL 33458 USA; ania.knapinska@fau.edu; 2 Department of Chemistry, The Scripps Research Institute/Scripps Florida, Jupiter, FL 33458, USA

**Keywords:** matrix metalloproteinase, extracellular matrix, cancer progression, immunosuppression, signal transduction, collagenolysis

## Abstract

For over 20 years, membrane type 1 matrix metalloproteinase (MT1-MMP) has been recognized as a key component in cancer progression. Initially, the primary roles assigned to MT1-MMP were the activation of proMMP-2 and degradation of fibrillar collagen. Proteomics has revealed a great array of MT1-MMP substrates, and MT1-MMP selective inhibitors have allowed for a more complete mapping of MT1-MMP biological functions. MT1-MMP has extensive sheddase activities, is both a positive and negative regulator of angiogenesis, can act intracellularly and as a transcription factor, and modulates immune responses. We presently examine the multi-faceted role of MT1-MMP in cancer, with a consideration of how the diversity of MT1-MMP behaviors impacts the application of MT1-MMP inhibitors.

## 1. Introduction

Membrane type 1 matrix metalloproteinase (MT1-MMP) was initially identified as a cell surface protease present in tumor cells [1]. Since then, MT1-MMP has become a highly sought after target in cancer therapy. The expression of MT1-MMP has been associated with poor prognosis in patients with melanoma, pancreatic cancer, advanced neuroblastoma, small cell and non-small cell lung cancer, mesothelioma, tongue squamous cell carcinoma, head and neck carcinoma, bladder cancer, breast cancer, colorectal cancer, and ovarian cancer [2,3,4,5]. Increased tumor cell expression of MT1-MMP enhances metastasis [6,7]. MT1-MMP induces the epithelial to mesenchymal transition (EMT) in prostate and squamous cell carcinoma cells [8,9]. MT1-MMP is needed for tumor cell transmigration through endothelium and basement membrane invasion [10]. Gliomas induce MT1-MMP expression and activity in microglial cells [11]. Cancer stems cells/tumor-initiating cells require MT1-MMP for growth, tumor initiation, invasion and metastasis, particularly in hypoxic, nutrient-deprived environments [12]. MT1-MMP is generally considered pro-invasive and pro-tumorigenic as (a) the expression and activity of MT1-MMP are elevated in tumor tissues and (b) high levels of MT1-MMP directly correlate with enhanced cell migration and tumor regional invasion/remote metastasis [13,14].

While extensive data indicates a significant role for MT1-MMP in cancer, studies of MT1-MMP have often focused on its activation of proMMP-2, hydrolysis of collagen, and shedding of CD44. Mass spectrometric analysis of biotin-labeled cell surface proteins revealed 158 binding partners for MT1-MMP [7]. MT1-MMP cell surface binding partners that have been validated include tetraspanins (CD9, CD37, CD53, CD63, CD81, CD82, CD151, and/or TSPAN12), the α2β1 and αvβ3 integrins, CD44, and a ternary complex with tetraspanins and the α3β1 integrin [7,15,16,17,18,19,20]. Proteomic approaches have uncovered a vast array of potential MT1-MMP substrates [21,22,23,24,25]. Advancements in bioanalytical methods have revealed that the precise behaviors of MT1-MMP that contribute to disease initiation and progression are now greater than believed even a few years ago [26,27,28].

## 2. Activities of MT1-MMP

**Figure 1:**
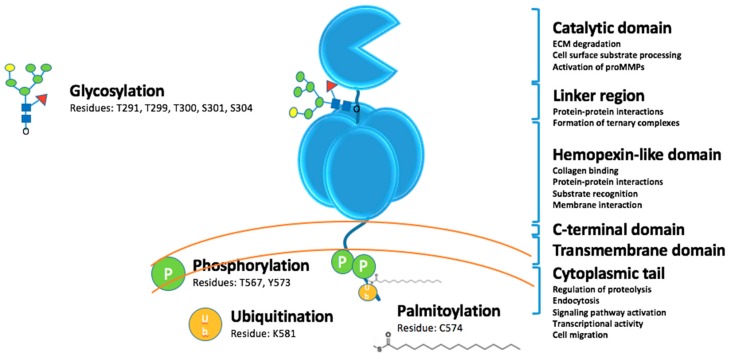
MT1-MMP domains and posttranslational modifications.

MT1-MMP functions on multiple levels in cancer growth and invasion (Figure 1). MT1-MMP can act in the following ways: (a) proteolysis of extracellular matrix (ECM) biomolecules, such as collagen, which allows for the activation of cell signaling pathways (based on the fragments generated by MT1-MMP action) and cell invasion through the ECM; (b) binding of ligands to MT1-MMP, which causes structural changes in MT1-MMP that affects interactions of MT1-MMP to cell surface partners and intracellular signaling of MT1-MMP via the cytoplasmic tail (CT); (c) intracellular proteolysis; and (d) as a transcription factor.

### 2.1. Extracellular Catalytic Activities

MT1-MMP was initially recognized for activating proMMP-2 [1,29,30,31,32,33]. MT1-MMP was subsequently shown to process types I, II, and III collagen and gelatin [33,34,35]. The combined action of MT1-MMP and MMP-2 was proposed to enhance ECM degradation and subsequent invasion [36,37]. Phagocytosis of collagen was found to be mediated by MT1-MMP, where the additional action of MMP-2 was not required [38,39]. MT1-MMP is the dominant collagenase for tumor invasion [40,41] and the proteolytic activity of MT1-MMP is critical for tumor cell invasion of three-dimensional (3D) collagen matrices [42,43,44,45,46]. Similarly, MT1-MMP promoted neovessel formation by facilitating endothelial cell invasion of collagenous matrices and tubulogenesis [47,48]. MT1-MMP is localized in invadopodia for ECM degradation and cell invasion [49,50].

In addition to proMMP-2, MT1-MMP can activate proMMP-13 [51]. In contrast, MT1-MMP processing of active MMP-11 inactivates the enzyme [52]. MT1-MMP has been shown to cleave and activate Notch1, leading to melanoma growth [53]. MT1-MMP activates the pro-αv integrin subunit, stimulating focal adhesion kinase (FAK) phosphorylation and cell migration on vitronectin [54]. MT1-MMP activates latent transforming growth factor-β (TGF-β) [55,56] and can release TGF-β by proteolytically processing the latent TGF-β binding protein (LTBP-1) [57]. MT1-MMP activation of TGF-β signaling induces the upregulation of CUTL1 and Wnt5a and ultimately EMT in prostate cancer cells [56]. MT1-MMP induction of EMT in squamous cell carcinoma was associated with increased levels of Twist, ZEB1, and ZEB2 and the repressed transcription of E-cadherin [9]. These activities were inhibited in the presence of a tissue inhibitor of metalloproteinase-2 (TIMP-2), but not TIMP-1, indicating that MT1-MMP catalytic activity was necessary [9].

CD44 binds to MT1-MMP via blade I of the HPX domain [3,15]. While MT1-MMP can cleave CD44 [58] and has been implicated for constitutive shedding of CD44 from the human melanoma cell surface [59], ADAM-10, MMP-9, and a chymotrypsin-like enzyme have also been described as CD44 sheddases [59,60,61,62]. In a similar fashion, both MT1-MMP and ADAM10 have been implicated in shedding DDR1 [63,64,65]. ADAM10 was shown to shed DDR1 upon collagen binding [65], regulating collagen-induced signaling in epidermoid carcinoma (A431), embryonic kidney (HEK293), and triple negative breast cancer (HC1806) cells. For these cell lines, shedding was insensitive to TIMP-2 or MT1-MMP knockdown [63,65], and thus MT1-MMP was not involved. In contrast, constitutive MT1-MMP-mediated DDR1 shedding was found to regulate collagen-induced signaling when DDR1 and MT1-MMP were co-expressed in COS1 cells, whereas MT1-MMP was suggested to be one of several DDR1 sheddases and regulators in HC1806 breast cancer cells [64].

MT1-MMP sheds protein-tyrosine kinase-7 (PTK7), a component of the Wnt/planar cell polarity pathway [66]. Shedding of PTK7 promoted tumor cell invasion [66]. MT1-MMP sheds mucin 16 (MUC16)/cancer antigen 125 (CA-125) from the surface of ovarian cancer cells [67]. This shedding reduces cell adhesion to mesothelial cells and may promote integrin-mediated adhesion and subsequent invasion [67]. MT1-MMP sheds extracellular matrix metalloproteinase inducer (EMMPRIN), where the released 22 kDa fragment may subsequently regulate MMP expression [68]. MT1-MMP hydrolysis of apolipoprotein E abrogates the suppression of cell proliferation [69].

MT1-MMP releases fibronectin from the cell surface [22]. MT1-MMP knockout mice have arrested tendon development [70]. Collagenolysis by MT1-MMP was not essential for tendon development, but MT1-MMP processing of fibronectin was, resulting in the release of fibrils from fibripositors [70]. MT1-MMP sheds death receptor-6 [22] and heparin-binding epidermal growth factor [71], in the latter case resulting in activation of the epidermal growth factor receptor. MT1-MMP sheds additional cell surface biomolecules, such as syndecan-1 [72], MHC class I chain-related molecule A (see below) [73], E-cadherin (see below) [74], low-density lipoprotein receptor-related protein 1 (LRP1/CD91) [75], mucin 1 [76], and tissue *trans*-glutaminase [77], and processes cytokines, chemokines, and growth factors, such as the pro-tumor necrosis factor [22]. The receptor Tyr kinase erythropoietin producing hepatocellular A2 (EphA2) is cleaved by MT1-MMP [78,79,80]. Cleavage at the Gly391-Leu392 bond promoted EphA2 internalization and single cell breast carcinoma invasion [78,79], while cleavage at Ser432-Tyr433 promoted ligand-independent activation of RhoG by EphA2 and epidermoid carcinoma cell migration [80].

The 30 kDa fragment released from MT1-MMP processing of the laminin-5 γ2 chain binds to the epidermal growth factor (EGF) receptor and stimulates cell migration [81]. It has been proposed that MT1-MMP processing of ECM components results in products binding to the β1 integrin, activation of the integrin leading to FAK phosphorylation at Tyr397, and the protection of tumor cells from chemotherapy- or radiotherapy-induced DNA damage [82]. Alternatively, MT1-MMP processing of collagen exposes RGD motifs [83,84], resulting in a shift from intact collagen binding to the α2β1 integrin to RGD-containing collagen fragments binding to the αvβ3 integrin [83]. This shift results in FAK phosphorylation at Tyr576 and Tyr577, the activation of ERK, and the promotion of cell migration [83]. ECM processing by MT1-MMP also promotes focal adhesion turnover, which facilitates migration [85]. MT1-MMP processing of type I collagen correlates with the repression of mature *let-7*, a tumor suppressive family of microRNAs, in pancreatic cancer [86].

Pancreatic ductal adenocarcinoma (PDAC) tumors increase the expression of MT1-MMP and TGF-β1 [87]. MT1-MMP processing of TGF-β results in the activation of this growth factor, increased collagen production by PDAC stellate cells, and hence an increased fibrotic microenvironment (“desmoplastic reaction”) [88]. Blocking the MT1-MMP function in 3D collagen gels sensitizes PDAC cells to gemcitabine [87,89]. This has been postulated as being due to products of MT1-MMP activity activating integrins and/or growth factor receptors and subsequent signaling enhancing ERK1/2 phosphorylation [87,89]. Over time, the increased production of collagen and TGF-β induces Smad3/4 and subsequently Snail, a regulator of EMT, in PDAC cells [87,90]. Snail then increases the expression of MT1-MMP, resulting in MT1-MMP-mediated PDAC cell invasion of collagen [90]. Snail1 increases the expression of MT1-MMP and breast carcinoma basement membrane invasion [91].

MT1-MMP extracellular activity can also inhibit cancer progression. MT1-MMP shedding of endoglin (CD105) results in the release of sEndoglin, which inhibits angiogenesis [92]. MT1-MMP sheds lymphatic vessel endothelial hyaluronan receptor-1 (LYVE-1) on lymphatic endothelial cells, inhibiting lymphangiogenesis and possibly lymphatic metastasis [93].

MT1-MMP is secreted in exosomes (extracellular vesicles) and is enzymatically active [94,95]. In rat models, pancreatic cancer-derived exosomes possess MT1-MMP, which then contributes to pre-metastatic niche formation [96].

MT1-MMP is glycosylated in the linker region between the CAT and HPX domains (Figure 1). Glycosylation can occur at Thr291, Thr299, Thr300, and Ser301, and Ser304 [97]. MT1-MMP may be differentially glycosylated in cancer cell lines [97,98,99]. Glycosylation does not impact zymogen activation, but does impact the interaction of MT1-MMP with TIMP-2 and the formation of the MT1-MMP•TIMP-2•proMMP-2 complex needed for proMMP-2 activation [98]. One report indicated that pericellular collagenolysis is not impacted by glycosylation [98], while another report came to the opposite conclusion [99]. It has been hypothesized that glycosylation may regulate TIMP-2-mediated endocytosis of MT1-MMP [98] and/or the conformation of MT1-MMP [99].

#### 2.1.1. The Role of MT1-MMP in Immunosuppression

MT1-MMP sheds tumor cell MHC class I chain-related molecule A (MICA) [73]. Engagement of MICA to NKG2D stimulates natural killer (NK) and T-cell antitumor activity [73]. Protection of MICA stimulated antitumor immunity and reduced metastasis in a humanized melanoma mouse model [100].

An MT1-MMP antibody, Fab 3369, reduced lung metastases following treatment of an MDA-MB-231 triple-negative breast cancer xenograft mouse model [101]. Examination of tumor cryosections revealed an increased density of iNOS+ cells (a marker of anti-tumor M1 tumor-associated macrophages) and Granzyme B+ cells [101]. The MT1-MMP antibody DX-2400, when applied in the 4T1 triple-negative breast cancer mouse model, inhibited tumor growth, shifted macrophages towards the antitumor M1-like phenotype, and reduced activated TGFβ (an immunosuppressive cytokine) [102]. TGF-β has been implicated as a signaling molecule produced by tumor cells that activates stromal cells [103] and, along with cancer ECM dysregulation, is associated with checkpoint (PD-1) blockade failure [104].

### 2.2. Intracellular Catalytic Activities

Subcellular mapping of the human proteome revealed that MT1-MMP is mainly localized to the cytosol and additionally to the intermediate filaments [105](http://www.proteinatlas.org/ENSG00000157227-MMP14/cell). MT1-MMP is trafficked along the tubulin cytoskeleton [106]. MT1-MMP is present in Rab-4-positive vesicles in the pericentrosomal compartment [107]. MT1-MMP exhibits several intracellular activities, including the cleavage of pericentrin (an integral centrosomal protein that coordinates the mitotic spindle) [106], the centrosomal breast cancer type 2 susceptibility gene (BRCA2) [108], metabolic enzymes (see below), and the cytoskeletal proteins ezrin and moesin [109].

**Figure 2:**
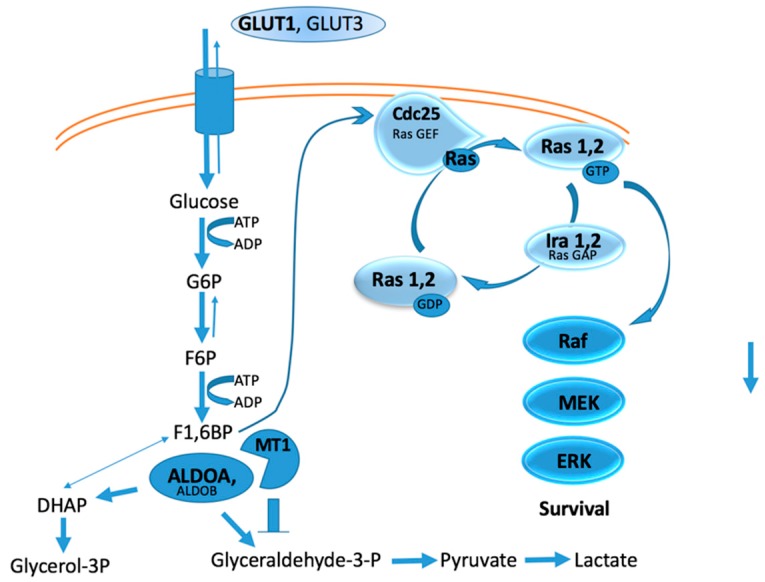
Hypothetical relationship between glycolysis, Ras activation, and MT1-MMP intracellular activity.

Deletion of MT1-MMP was found to correlate with changes in several metabolic pathways, where 142 proteins were significantly higher and 325 proteins significantly lower in MT1-MMP knockout tissue compared with wild-type tissue [24]. Glycogen synthase decreased while glycogen phosphorylase increased in MT1-MMP knockout tissue, resulting in decreased glycogenesis and increased glycogenolysis [24]. MT1-MMP intracellular substrates identified from cell-based proteomics include enolase-β, enolase-γ, fructose-bisphosphate aldolase A (ALDOA), glyceraldehyde 3-phosphate dehydrogenase (GAPDH), and phosphoglycerate phosphokinase 1 (PGK1) [109]. The above proteomic analysis revealed that ALDOA was significantly increased in MT1-MMP KO mice, suggesting that it is an in vivo substrate for MT1-MMP [24]. If MT1-MMP cleaved the above enzymes in tumor cells, glucose metabolism would be stopped at the fructose-1,6-bisphosphate (F1,6BP) stage (Figure 2). One result would be that the use of glucose shifted to the pentose phosphate pathway, hexosamine synthesis pathway, and glycogenesis [110]. A second, and perhaps more significant, result, would be enhanced Ras activation, as F1,6BP can activate Ras by acting through Cdc25 (Figure 2) [111]. The F1,6BP/Ras relationship establishes a link between glycolysis and cell proliferation [111]. Thus, MT1-MMP intracellular activity could further enhance Ras activation (Figure 2).

### 2.3. Signaling Activities

Posttranslational modification of the MT1-MMP CT (Figure 1) promotes tumor cell proliferation and invasion and tumor growth [112,113,114]. For example, LIM kinase-1 (LIMK1) phosphorylates Tyr573 in the MT1-MMP CT [115]. LIMK1 interaction with MT1-MMP modulates the catalytic activity of the enzyme [115]. Src-dependent phosphorylation of Tyr573 promotes the formation of a FAK•p130Cas•MT1-MMP complex, which facilitates tumor cell degradation of ECM at focal adhesion sites [116]. In contrast, Src-dependent phosphorylation of Tyr573 has been reported to impact tumor cell migration and proliferation, but not MT1-MMP catalytic activities [112,117]. Epidermal growth factor-induced phosphorylation of Tyr573 results in the internalization of MT1-MMP and expansive ovarian carcinoma cell growth [118]. Phosphorylation at Tyr573 was found to be a prerequisite for ubiquitination [119]. Mono-ubiquitination at Lys581 in the CT was catalyzed by the E3 ubiquitin-protein ligase NEDD4 [119]. A lack of ubiquitination resulted in reduced cell surface levels of MT1-MMP and increased localization in endosomes [119].

Phosphorylation of CT Thr567 regulates MT1-MMP shedding of the α3 integrin ectodomain in ovarian carcinoma [113]. PKC-mediated Thr567 phosphorylation increased breast cancer cell type I collagen and Matrigel invasion and growth within a 3D collagen matrix [113]. Phosphorylation of the MT1-MMP CT Thr567 enhances ovarian cancer aggregation (spheroid formation) by minimizing MT1-MMP shedding of E-cadherin [114]. Palmitoylation of Cys574 facilitates the internalization of MT1-MMP by the clathrin-dependent pathway [120].

The MT1-MMP CT stimulated aerobic glycolysis (and ATP production) by increasing the expression of hypoxia-inducible factor 1α (HIF-1α) target genes [121,122]. More specifically, Factor Inhibiting HIF-1 (FIH-1) binds to the MT1-MMP CT, directing FIH-1 to interact with Mint3 and deterring FIH-1 repression of HIF-1 transcriptional activity [28,122]. Thus, under normoxia, aerobic glycolysis (the Warburg effect) occurs, accompanied by active HIF-1 [28,122]. HIF-1 increases the expression of glucose transporter 1 (GLUT1), hexokinase 2 (HK2), lactate dehydrogenase (LDHA), and monocarboxylate transporter 4 (MCT4) [110]. The overall result is more glucose coming into the cell, more conversion of glucose to pyruvate and then to lactate, and more lactate secretion from the cell [110]. Inhibiting the CT interactions of MT1-MMP decreased lactate production and tumor growth [122].

MT1-MMP stimulated melanoma motility by signaling independent of catalytic activity [123]. The Ras/Raf/ERK1/2 signaling cascade is induced upon low, physiological levels of TIMP-2 binding to MT1-MMP and promotes cell migration and tumor growth [9,124]. TIMP-2 also promotes signaling in the catalytically inactive mutant of MT1-MMP, and pathway induction is based on TIMP-2 binding to the HPX domain of MT1-MMP [124]. The growth of tumor xenografts expressing wild-type or catalytically inactive MT1-MMP greatly exceeded that of tumors that expressed no MT1-MMP [9,124]. Additional studies support the notion that cell migration may not require catalytic activity or the CT, and may be due to HPX domain interactions with cell surface binding partners [125]. The MT1-MMP CT is required for concanavalin-A-induced autophagy in glioblastoma cells [126]. Ultimately, by associating with cell surface ECM receptors, receptor Tyr kinases, and tetraspanins via ectodomains, and intracellular signaling proteins via the CT, MT1-MMP can remodel the ECM and promote signaling [26]. In contrast, increased COS-7 cell migration via ERK activation required catalytic activity and the CT of MT1-MMP [127].

MT1-MMP catalytic activity was required for mammary epithelial cells branching in dense but not sparse three-dimensional collagen gels [128]. In comparison, a non-proteolytic function of MT1-MMP was found to be required for branching in both dense and sparse conditions [128]. MT1-MMP directly associated with the β1 integrin subunit through the MT1-MMP transmembrane domain and CT, and this interaction modulated the β1 integrin-dependent signals that mediated mammary epithelial cell invasion during branching morphogenesis [128].

MT1-MMP•CD44 association leads to localization to lamellipodia [15,129]. The interaction of MT1-MMP with CD44 promotes signaling through EGFR activation to the MAPK and PI3K pathways, enhancing cell migration [3]. The cytoplasmic tails of MT1-MMP and CD44 can simultaneously bind to the FERM domain of radixin [130]. Radixin interacts with the region spanning residues 566-576 of the MT1-MMP CT [130].

Interaction of MT1-MMP cytoplasmic tail binding protein 1 (MTCBP-1) with MT1-MMP displaces the enzyme from invadopodia by disrupting the interaction of the CT Leu-Leu-Tyr region (residues 571-573) with F-actin [131]. This in turn reduces pancreatic cancer metastasis [131].

### 2.4. Transcription Regulatory Activities

MT1-MMP regulation of transcriptional programs has been demonstrated in a number of cell lines [121,132]. Overexpression of MT1-MMP increased the transcription of vascular endothelial growth factor A (VEGF-A) in MCF-7 and U251 cells and, concurrently, tumor growth, angiogenesis, and metastasis [54,133]. Transcription of VEGF-A was regulated through MT1-MMP catalytic activity and the CT, as well as Src kinase activity [132]. MT1-MMP regulated the transcription of dickkopf-related protein 3 (DKK3) in urothelial cells and Smad1 in several tumor cell lines [132]. In phorbol-12-myristate-13-acetate (PMA)-stimulated HT1080 cells, the expression of MT1-MMP modulated inflammasome gene expression [134]. The transcription of IL-33 and IL-12A was MT1-MMP-dependent [134]. MT1-MMP was found to translocate to the nucleus, where it induced the expression and activation of the phosphoinositide 3-kinase δ/Akt/GSK3β signaling cascade [135]. Induction of this cascade modulated macrophage immune responses [135,136]. MT1-MMP catalytic activity decreases the expression of the tumor suppressor SPRY4 in metastatic melanoma through an MMP-2/RAC1 pathway; a higher expression of SPRY4 correlated with a longer survival of melanoma patients [137].

## 3. Overview

The initial view of the role of MT1-MMP in cancer progression was straightforward: activation of proMMP-2 and degradation of fibrillar collagen to facilitate metastasis. The contributions of MT1-MMP to cancer progression are now viewed as far more complex based on the number of MT1-MMP substrates identified. MT1-MMP activity has a negative impact on immune responses to tumors, and intracellular MT1-MMP activity regulates cancer cell metabolic functions. MT1-MMP has a significant role in angiogenesis, whereby it can exhibit both pro-angiogenic and anti-angiogenic behaviors [19,47,48,138,139,140,141,142,143]. These contrasting behaviors point to the importance of the spatial and temporal expression of MT1-MMP. Active MT1-MMP has been found to be highly expressed in stromal cells of the tumor microenvironment (cancer-associated fibroblasts, macrophages, etc.) rather than the tumor epithelium in mouse models of pancreatic and breast cancer [103,131]. Thus, there are considerations as to how the tumor induces MT1-MMP production. The tumor microenvironment also impacts MT1-MMP activity based on the local pH and oxygen and nutrient content.

Several creative strategies have led to the development of highly selective MT1-MMP activity inhibitors [144,145,146]. Of particular interest would be approaches that avoid active site targeting of MT1-MMP, in consideration of prior failures of active site targeting MMP inhibitors in clinical trials. Numerous antibodies have been described that modulate MT1-MMP proteolytic activity by interacting with secondary binding sites (exosites) [144,145,146]. In a similar fashion, the compound NSC405020 [3,4-dichloro-*N*-(1-methylbutyl)benzamide] was found to bind to the MT1-MMP HPX domain, inhibit MT1-MMP homodimerization, and reduce tumor size significantly in mouse models [147]. Inhibitors could be designed to disrupt cell surface complexes, such as MT1-MMP association with tetraspanins, the α2β1 and αvβ3 integrins, CD44, and the ternary complex with tetraspanins and the α3β1 integrin. Peptide IS4 (acetyl-VMDGYPMP-NH_2_), modeled on the region of the MT1-MMP HPX domain that binds CD44 (the outermost strand of blade I), inhibited MT1-MMP-mediated cell migration and metastasis in vivo [125]. CT interactions of MT1-MMP can be inhibited using a peptide model (7R)-CPT (RRRRRRRGRRHGTPRRLLYCQRSLLDKV), resulting in decreased tumor growth [122]. Inhibitors of signaling pathways that impact MT1-MMP function can also be utilized to modulate the enzyme. In order to impact cancer in a positive way, the successful application of these inhibitors will require a thorough consideration of mode of administration (systemic versus topical), mechanism of action (extracellular versus intracellular), cancer stage (pre-metastatic versus metastatic), and potential side effects. It is worth noting that the inhibition of MT1-MMP activity in triple-negative breast cancer mouse models improved tumor profusion and sensitized the tumor to ionizing radiation or doxorubicin treatments [82,102].
